# Plain X-ray is insufficient for correct diagnosis of tibial shaft spiral fractures: a prospective trial

**DOI:** 10.1007/s00068-023-02285-x

**Published:** 2023-06-03

**Authors:** Leonard Lisitano, Timon Röttinger, Andreas Wiedl, Kim Rau, Sönke Helling, Jairo Cifuentes, Bertram Jehs, Mark Härting, Laura-Marie Feitelson, Johannes Gleich, Sophia Kiesl, Daniel Pfeufer, Carl Neuerburg, Edgar Mayr, Stefan Förch

**Affiliations:** 1 Department for Trauma, Orthopedics, Hand and Plastic Surgery, University Hospital Augsburg, Stenglinstr. 2, 86156 Augsburg, Germany; 2 Department for Diagnostic and Interventional Radiology, University Hospital Augsburg, Augsburg, Germany; 3 Department for Orthopedics and Trauma Surgery, Musculoskeletal University Center Munich (MUM), University Hospital LMU Munich, Munich, Germany; 4 Department of Radiology, University Hospital LMU Munich, Munich, Germany

**Keywords:** Tibia fractures, Diagnostic errors, Delayed diagnosis, Ankle fractures

## Abstract

**Purpose:**

Tibial shaft spiral fractures and fractures of the distal third of the tibia (AO:42A/B/C and 43A) frequently occur with non-displaced posterior malleolus fractures (PM). This study investigated the hypothesis that plain X-ray is not sufficient for a reliable diagnosis of associated non-displaced PM fractures in tibial shaft spiral fractures.

**Methods:**

50 X-rays showing 42A/B/C and 43A fractures were evaluated by two groups of physicians, each group was comprised of a resident and a fellowship-trained traumatologist or radiologist. Each group was tasked to make a diagnosis and/or suggest if further imaging was needed. One group was primed with the incidence of PM fractures and asked to explicitly assess the PM.

**Results:**

Overall, 9.13/25 (SD ± 5.77) PM fractures were diagnosed on X-ray. If the posterior malleolus fracture was named or a CT was requested, the fracture was considered “detected”. With this in mind, 14.8 ± 5.95 posterior malleolus fractures were detected.

Significantly more fractures were diagnosed/detected (14 vs. 4.25/25; *p* < 0.001/14.8 vs. 10.5/25; *p* < 0.001) in the group with awareness. However, there were significantly more false positives in the awareness group (2.5 vs. 0.5; *p* = 0.024). Senior physicians recognized slightly more fractures than residents (residents: 13.0 ± 7.79; senior physicians: 16.5 ± 3.70; *p* = 0.040). No significant differences were demonstrated between radiologists and trauma surgeons.

The inner-rater reliability was high with 91.2% agreement. Inter-rater reliability showed fair agreement (Fleiss-Kappa 0.274, *p* < 0.001) across all examiners and moderate agreement (Fleiss-Kappa 0.561, *p* < 0.001) in group 2.

**Conclusion:**

Only 17% of PM fractures were identified on plain X-ray and awareness of PM only improved diagnosis by 39%. While experiencing improved accuracy, CT imaging should be included in a comprehensive examination of tibial shaft spiral fractures.

**Level of evidence:**

II. Diagnostic prospective cohort study.

**Trail registration number:**

DRKS00030075.

## Background

Concomitant fractures of the posterior malleolus (PM) typically occur in distal tibia fractures (Arbeitsgemeinschaft für Osteosynthesefragen Classification; AO:43A) and especially in spiral fractures of the tibial shaft (AO:42/43A) with a fracture line extending from proximal-lateral to distal-medial in the anterior–posterior (AP) view (type A). The incidence of concomitant PM fractures in tibial shaft fractures type A has been cited to be greater than 50%, and importantly, not all of them were detected preoperatively [1].

Although the occurrence of PM fractures in tibial shaft spiral fractures is now well established, there is no evidence that plain X-ray imaging is sufficient to rule out additional PM fractures. This is clinically relevant—especially for tibial shaft fractures with a high risk of additional PM fractures—to avoid unnecessary radiation, but at the same time not to overlook any PM fractures. Missed PM fractures can lead to intraoperative complications such as secondary dislocation as well as postoperative instability and post-traumatic osteoarthritis. Fractures with an increased risk of PM involvement have already been described several times in the literature [2–4].

The management of fractures of the PM is currently changing. Several recent publications suggest that surgical fixation—even of small fragments—is important for restoring the articular surface, the fibular notch and trans-syndesmotic stability [5]. Therefore, CT imaging is necessary to visualize the fragment in size, comminution and articular impaction and for preoperative planning [5, 6]. This is important, as several studies on ankle fractures have shown that plain radiographs are not sufficient to provide surgically relevant information [7, 8]. While these studies were on ankle fractures, there may be a similar issue with PM fractures accompanying tibial shaft fractures.

This study investigated the hypothesis that plain X-ray is not sufficient for a reliable diagnosis of associated non-displaced PM fractures in tibial shaft spiral fractures.

## Material and methods

50 plain X-rays of patients from the emergency department (ER) of a Level I Trauma Center with tibial shaft fractures (AO:42A/B/C and 43A) were prospectively evaluated by different physicians. 25 of the 50 patients had an additional fracture of the posterior malleolus. To avoid a selection bias, the last 25 patients admitted to the ER at a Level I Trauma Center meeting the inclusion criteria were used as the study sample for each group (25 tibial shaft fractures with additional PM fractur and 25 tibial shaft fractures without additional fractures; date of data collection: April 1, 2021). Mean age was 47.1 ± 20.0 years old (min. 18, max. 92).

All X-rays were initially screened by a specialist in trauma surgery and a specialist in radiology, who independently assessed the images based on the inclusion and exclusion criteria. Tibial shaft fractures were included if there was no direct joint involvement and both plain X-ray and CT imaging of the ankle were available. Fractures without direct joint involvement were defined as all fractures where the main/obvious fracture does not affect a joint. CT imaging was used to confirm the presence of a PM fracture. If both specialists agreed, the diagnosis (PM fracture or no PM fracture) was regarded as certain and used as a basis for the subsequent evaluation of the plain X-ray images. Pathological fractures, open fractures, multiple trauma, imaging of insufficient quality and children under 18 years of age were excluded. The patient data (name, date of birth) were irreversibly anonymized and replaced by numerical codes on all research materials.

The selected plain X-rays (at least one AP and one lateral view of sufficient quality) were evaluated by two groups of physicians. Each group consisted of a radiological resident and a senior physician, as well as a trauma surgery resident and a senior physician. All residents had at least three years of professional experience. The assessing physicians did not have access to the CT imaging. All examinations were carried out at the physicians' daily workplaces.

The first group (group 1) was unaware of the previous study describing the incidence of concomitant posterior malleolus fractures in type A fractures as the analysis was carried out before the study results were published (January 2022). All physicians were asked to provide a full diagnosis for all patients and to request further imaging if necessary. Only the trauma surgeons were specifically tasked with recommending appropriate treatment options in addition.

The second group (group 2) was explicitly informed of the results of the preliminary studies and asked explicitly about a fracture of the posterior malleolus. Again, this group could request additional imaging if necessary and the surgeons could suggest treatment options. In this group, the examination of the same radiographs was repeated in a different order after 4–6 weeks.

To avoid bias, all physicians were only asked for additional CT-imaging necessary to make the diagnosis, not for surgical planning.

The primary outcome was the correct diagnosis of accompanying PM fractures in plain X-rays and the secondary outcome was the preoperative detection of accompanying PM fractures. A PM fracture was considered as detected if it was diagnosed in plain X-ray or a CT showing the ankle was requested or both (CT requests for surgical planning were not counted).

All statistical testing was carried out with Jamovi 2.2.5 (jamovi.org) and for Fleiss’ kappa with SPSS 28 (IBM Germany). Descriptive statistics were presented as mean ± standard deviation. The *t* test for dependent samples and the Wilcoxon rank test were used for the significance tests. *p* ≤ 0.05 was set as the significance level.

Fleiss’ kappa and Cohen’s kappa are both statistical measures used to assess the level of agreement between multiple raters. [9, 10] In this study, these kappa values were employed to measure the agreement between the different physicians involved in the assessment or diagnosis process. Both kappa values range from − 1 to 1, where a value of 1 indicates perfect agreement, 0 implies agreement is no better than chance, and − 1 signifies perfect disagreement.

The study was performed in accordance with the ethical standards laid down in the 1964 Declaration of Helsinki and its later amendments. The use of patient data was allowed by the local Ethics Committee.

## Results

Overall, an average of 9.13 ± 5.77 of the 25 (36.5%) posterior malleolus fractures were correctly diagnosed. In group 1 (no awareness) 4.25 ± 3.77, in group 2 (increased awareness) 14 ± 0 were diagnosed. This was statistically significant (*p* < 0.001, post-hoc power 83.9%). However, there were statistically significant more false positives in group 2 (*p* = 0.024). Among the fractures without an additional posterior malleolus fracture, on average 2.5 ± 1.29 of 25 patients were misdiagnosed with a posterior malleolus fracture in group 2. In group 1 the average was 0.5 ± 0.5.

Sensitivity for detecting additional PM fractures in plain X-rays was 0.17 in group 1 and 0.56 in group 2 with a specificity of 0.98 (group 1) and 0.90 (group 2; overall sensitivity 0.365, specificity 0.94) (see Table 1).

**Table 1:** Summary of PM fractures diagnosed in plain X-rays

	All examiners	Group 1 (no awareness)	Group 2 (awareness)	*p* value (Group 1 vs. Group 2)
Diagnosed PM fractures (X-ray)	9.13 ± 5.77/25	4.25 ± 3.77/25	14 ± 0/25	< 0.001**
False positive (no PM fracture)	1.5 ± 0.645/25	0.5 ± 0.5/25	2.5 ± 1.29/25	0.024*
Sensitivity for diagnosing PM fractur in X-ray	0.365	0.17	0.56	N/A
Specificity for diagnosing PM fractur in X-ray	0.94	0.98	0.90	N/A

“Diagnosed PM fractures” refers to the 25 patients with addition PM fracture in the CT imaging. “False positive” refers to the 25 patients without a PM fractur in the CT imaging. **Indicates highly significant values. *Indicates significant values

Preoperative detection of additional fractures of the posterior malleolus is clinically relevant. In the following, a fracture of the posterior malleolus was considered "detected" if it was either diagnosed by plain X-ray or a CT showing the ankle joint was requested based on the available X-ray (or both). Due to the high sensitivity of CT imaging, it was assumed that all additional fractures of the PM would have been detected on CT.

Considering this, an average of 14.8 ± 5.95 of 25 posterior malleolus fractures were detected. Statistically significantly more additional PM fractures were detected in group 2 (increased awareness) (10.5 ± 5.80 in group 1 vs. 19.0 ± 0.82 in group 2; *p* < 0.001). A maximum (best examiner) of 20/25 fractures and a minimum (worst examiner) of 4/25 fractures were detected by the individual examiners (see Table 2).

**Table 2:** Summary of the additional PM fractures that would have been detected before surgery (diagnosed fractures in plain X-ray or (/and) for diagnostic purpose requested CT imaging showing the ankle joint based on the plain X-ray)

	All examiners	Group 1 (no awareness)	Group 2 (awareness)	*p* value (Group 1 vs. Group 2)
Preoperatively detected PM fractures	14.8 ± 5.95/25	10.5 ± 5.80	19.0 ± 0.82	< 0.001**
Interrater reliability (Fleiss’ Kappa)	0.274 (*p* < 0.001)	0.234 (*p* = 0.004)	0.561 (*p* < 0.001)	N/A

**Indicates highly significant values

Senior physicians recognized slightly more accompanying PM fractures than residents (residents: 13.0 ± 7.79; senior physicians 16.5 ± 3.70; *p* = 0.040). No significant differences in detected fractures could be demonstrated between trauma surgeons and radiologists (Trauma surgeons 14.8 ± 7.18; radiologists 14.8 ± 5.56; *p* = 0.644) (see Table 3).

**Table 3:** Summary of the additional PM fractures that would have been detected before surgery divided by level of training and specialty

	All examiners	Radiologists	Trauma surgeons	*p* value
Preoperatively detected PM fractures	14.8 ± 5.95/25	14.8 ± 5.56/25	14.8 ± 7.18/25	0.644

	All examiners	Residents	Specialists	*p* value
Preoperatively detected PM fractures	14.8 ± 5.95/25	13.0 ± 7.79/25	16.5 ± 3.70/25	0.040*

* indicates significant values

Table 4 shows an overview of the additional PM fractures detected on plain X-ray in the 25 patients with CT-confirmed PM fracture (CT imaging was not available for the examiners).

**Table 4:**
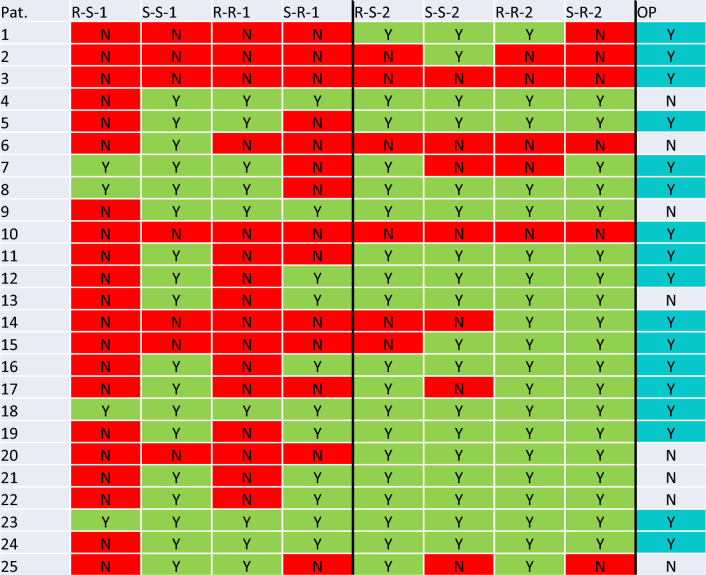
Overview of patients (#1–25; first column) with additional fractures of the posterior malleolus

The last column indicates whether the fracture of the posterior malleolus was surgically stabilized (screw fixation; Y = Yes; N = No). The remaining columns show the individual physicians results in the first survey. Y = posterior malleolus fracture detected, i.e. the fracture was described or CT imaging showing the ankle was requested. N = the fracture was not named and no CT imaging was requested. R-X-X = resident; S-X-X = senior physician; X-S-X = surgery; X-R-X = radiology; X-X-1 = group 1; X-X-2 = group 2

The inner-rater-reliability (the same examiner assesses the same X-ray images a second time) for detection of an additional fracture in group 2 from the first to the second assessment showed a high absolute agreement of 91.2% in average (max. 100%, min. 76%). Cohen’s kappa was calculated for all examiners and showed “moderate” to “almost perfect” agreement (0.534–0.907; *p* < 0.002). [11]

The inter-rater-reliability for all physicians (8 examiners) for the first review indicated “fair” agreement for diagnosed (Fleiss’ kappa = 0.292, *p* < 0.001) and detected (Fleiss’ kappa = 0.274, *p* < 0.001) additional posterior malleolus fractures.

Divided into groups, group 1 (no previous knowledge) showed a fair agreement (Fleiss’ kappa = 0.234, *p* = 0.004), and group 2 (with previous knowledge) a moderate agreement (Fleiss’ kappa = 0.561, *p* < 0.001) for the different observers.

## Discussion

The results show that based on plain X-rays only it was not possible to rule out accompanying PM fractures in tibial shaft fractures with certainty. Although increased awareness led to a significant improvement in sensitivity (0.17 vs. 0.56), it was still not possible to achieve the required reliability for responsible patient treatment.

**Fig. 1:**
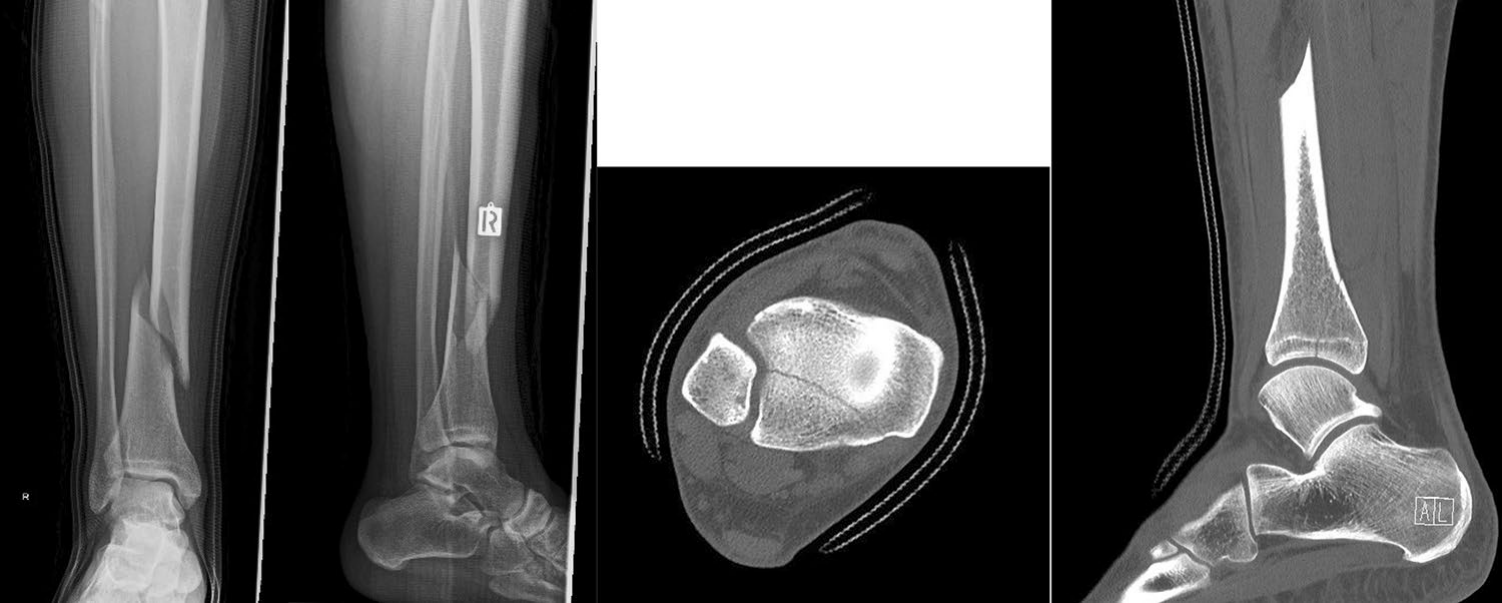
The figures show an example of the X-ray and CT images of a patient with a tibial shaft spiral fracture type A with fracture of the posterior malleolus. CT images were not available for the examiners. Additional screw osteosynthesis of the posterior malleolus was performed in this patient

There are several studies indicating high incidences of additional PM fractures in tibial shaft fractures (25–50%) [11–13]. A large meta-analysis on PM fractures in tibial shaft fractures identified an incidence rate of 70% when utilizing CT or MRI, however, half of these were occult on plain X-ray (Fig. 1) [12].

No study has dealt with the reliability of X-ray in identifying these fractures and investigated the impact of previous knowledge, awareness, specialization and level of training on diagnostic accuracy. The present study closes this gap in the literature and shows that plain X-ray imaging is not sufficient to ensure a reliable diagnosis of tibia shaft fractures with a high risk of associated PM fractures—regardless of the examiner. Even with advanced awareness of additional PM fractures, 20% (5/25) of the additional PM fractures in tibial shaft spiral fractures—especially in type A fractures [1]—were not recognized preoperatively. This is clinically highly relevant as treatment of any fracture relies on a correct and complete diagnosis, which could not be achieved with plain X-ray diagnostics only.

Fractures with a high risk of PM involvement are primarily spiral fractures and fractures in the distal third of the tibia [1, 2, 4]. Both Marchand et. al. as well as Fisher et al. demonstrated this in similar studies. While Fisher et. al. identified spiral fractures, fractures in the distal third and fractures with an angle > 45° as independent risk factors for additional PM fractures, Marchand et. al. use the ratio of fracture length to distance to tibial plafond to identify fractures with a low risk of joint involvement (negative predictive value of 100% with ratio < 0.224) [2, 4].

Bouche et. al. compared the detection rates of PM fractures in bimalleolar fractures on plain X-ray or CT in a retrospective study. In this study, both, the plain X-rays and the CT scans, were evaluated by 2 surgeons for the presence of a PM fracture twice with an interval of 6 weeks. Similar to the present study, significantly fewer PM fractures were detected on X-rays (35/60 in X-ray vs. 53/60 in CT) and the interrater-reliability on plain X-rays was in a comparable range with a kappa of 0.39 (0.292 in our study). These results support the present results of the current study in that PM fractures cannot be excluded with certainty in the plain X-ray. However, compared to the present study, the study refers to ankle fractures and there were fewer examiners (2 vs. 8) [13].

Furthermore, there is further evidence that the size of the fragments in PM fractures cannot be adequately judged on plain X-rays and that there is poor interrater reliability for these fractures (in plain X-rays) [8, 14]. According to Solan/Sakellariou CT imaging is mandatory for the assessment of PM fractures and even for fractures that are only suspicious for an involvement of the PM [15].

There are no standardized treatment guidelines for fractures of the PM, however, the size of the PM has been a classic indication of internal fixation. Recent studies suggest that other fracture factors may be more important clinically [6, 16, 17]. A meta-analysis on ankle fractures found that fracture displacement, congruency of the articular surface, and residual tibiotalar subluxation were more relevant for the outcome of PM fractures than the fragment’s size [16]. Preoperative cross-sectional imaging is required for a precise assessment of these factors. Despite the increasing number of publications on PM fractures, there is no consensus on therapy yet [17].

In the patient population of the present study, in which preoperative CT imaging was available for all patients, all PM fractures large enough for screw osteosynthesis were fixated internally. Of these fractures, there were 2/17 fractures requiring surgical fixation that would not have been recognized preoperatively by all 8 experienced examiners in their daily routine (see Table 1 #3 and #10).

In addition, the presence of a fracture of the posterior malleolus is essential for surgical planning as the insertion of an intramedullary nail can result in secondary dislocation of the posterior malleolus. To avoid this, it must either be fixed with screw osteosynthesis beforehand or plate osteosynthesis must be used instead of the intramedullary nailing.

Despite the known accumulation of PM fractures in tibial shaft spiral fractures type A and distal tibial fractures, the indication and planning of the surgery are usually carried out without CT imaging on the basis of plain X-rays. Therefore it can be assumed that the PM fractures that are overlooked and not treated in everyday care occur even more frequently here than in ankle fractures, where preoperative CT scans are more common. Due to the ongoing trend towards earlier mobilization and weight bearing in recent years, the reliable detection and surgical fixation of additional PM fractures is becoming increasingly important [18–21]. While an non-displaced additional PM fracture may heal adequately within 6 weeks of non-weight bearing, the risk of secondary dislocation of undetected fractures increases significantly with immediate full weight bearing.

The present study has some limitations. There are previous publications demonstrating the coincidence of PM fractures in tibial shaft fractures, so the “no awareness” group maybe had some awareness for PM fractures [2, 22–25]. The limited sample size of 50 patients can potentially affect the generalizability of the results and may raise concerns about the study's statistical power. However, despite the modest number of patients, the findings demonstrated statistical significance, supporting the validity of the conclusions drawn from the study. Moreover, a post hoc power analysis for the primary outcome (correct diagnosis of accompanying PM fractures in plain X-rays) revealed a power of over 80%, which further strengthens the reliability of the results. Additionally, the screening of patients for inclusion and exclusion criteria may introduce a selection bias. To avoid this potential bias, the screening process was independently performed by a specialist in trauma surgery and a specialist in radiology. Another possible limitation is the variability in the indication for surgery among different surgeons, as there is no consistent definition of which PM fragments can be grasped with screw osteosynthesis.

However, in our opinion, these limitations do not affect the key statement, that plain X-ray imaging is not sufficient for comprehensive diagnosis of accompanying PM fractures in tibial shaft fractures.

The strengths of the study are its prospective design, the inclusion of radiologists and trauma surgeons, and the comparison of different levels of training. In conjunction with the incidences of fractures of the posterior malleolus in tibial shaft fractures described in the literature, we recommend preoperative CT imaging for all tibial shaft spiral fractures—especially with a fracture path in the AP X-ray extending from proximal-lateral to distal-medial (type A)—and all tibial fractures in the distal third.

## Conclusion

Concomitant fractures of the posterior malleolus in tibial shaft fractures were not reliably detected in plain X-rays, regardless of physicians’ specialty or level of training. Awareness of the frequency of these additional fractures in tibial shaft spiral fractures with a course from proximal-lateral to distal-medial in the AP X-ray (type A) leads to a significantly higher detection rate, but also to a more frequent misdiagnosis in the absence of a fracture of the posterior malleolus. None of the investigators could reach a satisfactory level of certainty in the detection of concomitant posterior malleolus injuries in plain X-rays. Consequently, plain X-ray imaging is not sufficient for the diagnosis of tibial shaft spiral fractures.

## Data Availability

The datasets used and analysed during the study are available from the corresponding author on reasonable request.

## Abbreviations

AP X-ray: Anterior–posterior X-ray
CT: Computed tomography
ER: Emergency room
max.: Maximum
min.: Minimum
PM: Posterior Malleolus
vs.: Versus
